# Transient Electromyographic Responses by Isokinetic Torque Release during Mechanically Assisted Elbow Flexion

**DOI:** 10.5114/jhk/169368

**Published:** 2023-10-11

**Authors:** Jeewon Choi, Ping YeapLoh, Satoshi Muraki

**Affiliations:** 1 Department of Industrial and Management Systems Engineering, Dong-A University, Busan, South Korea.; 2 Faculty of Design, Kyushu University, Fukuoka, Japan.

**Keywords:** power assistance, torque variability, rotation speed, dynamometer system, perceived assistance

## Abstract

Power assistance on joint torque may not be beneficial to all the related muscles. We investigated the effects of power assistance on torque release during isokinetic elbow flexion. An isokinetic dynamometer system was used to simulate dynamic elbow flexion with power assistance, which altered the exercise conditions of baseline isometric torque (greater and lower) and rotation speed (faster and slower) of the lever arm. Ten male right-handed participants performed exercise tasks using the system. We measured (1) the electromyogram (EMG) amplitudes of the biceps brachii (BB), brachioradialis (BR), and triceps brachii (TB) muscles, (2) torque output and its variability, and (3) the perceived assistance level. Transient responses of the objective measurements were analyzed by observing three time epochs before and after power assistance. Greater variability and lower perceived assistance levels were observed when greater torque was released at a faster rotation speed. The torque output and EMG amplitudes of BB and BR muscles decreased over time. However, EMG amplitudes in the TB muscle were relatively constant until 200 ms after power assistance resulting in greater muscle co-contraction. This could be attributed to the increased postural stability of the human musculature system when the external perturbation on joint movement occurred by power assistance, independent of exercise conditions.

## Introduction

Humans coordinate body movements by generating and releasing joint torque. The magnitude of the generated joint torque is dependent on the activation of the corresponding muscles required for the movement (Buchanan et al., 1989). A recent collaboration between humans and newly developed wearable power assistance devices has demonstrated the ability to augment joint torque and partially replace the role of muscles (Huysamen et al., 2018; Lee et al., 2012). This collaboration is expected to decrease the load required for many human activities, including upper limb movements required during tasks of daily living and in labor-intensive industrial fields (Galle et al., 2017; Iranzo et al., 2020; Papla et al., 2023; Sylla et al., 2014). However, users may not be easily able to anticipate how different torque and speed will be generated by the device. Although some upper-limb power assistive robots utilize electromyography (EMG) signals to estimate the user's intended motion torque (Kiguchi et al., 2004; Peternel et al., 2016), there could be a potential gap between how a user plans movement and how it is precisely reflected in the EMG signals. Thus, it has not yet been established how humans instantly react to external power assistance. This could potentially influence smooth adaptation to this collaboration in the long term.

Collaboration with power assistance could be more unsuited to upper limb movements because while typical movements such as lifting and moving objects increase tension on the agonist muscles, power assistance helps to release joint torque generated by the agonist muscles (Novakovic and Sanguineti, 2011). Compared with the motor unit activation strategy for muscle contraction and joint torque development, the deactivation strategy for muscle relaxation has greater uncertainty (Andrzejewska et al., 2014). Greater variability was also observed during linear torque release in an isometric contraction (Choi et al., 2019; Orizio et al., 2010). Previous studies that simulated power assistance on isometric elbow flexion have demonstrated that a slow force releasing rate reduces force variability, and greater change of force magnitude results in high variability while target overshoots decrease by adopting a conservative control strategy (Choi et al., 2020a, 2019). Specifically, the role of the agonist muscle has been highlighted during isometric exercise, as it tries to stabilize motor performance when power assistance is provided and causes external perturbation of the elbow joint.

In order to stabilize the joint movement against such an external perturbation, the musculature system increases the co-contraction of antagonist muscles, which results in increased joint stiffness (Latash, 1992; Lewis et al., 2010). The magnitude and direction of perturbation influence the level of relevant muscle activation (Franklin et al., 2003; Holmes and Keir, 2012). However, the directions of perturbation studied previously has continuously deviated from the intended direction of movement. Herein, we investigated how these joint stabilizing mechanisms functioned when power assistance was provided in the same direction as elbow flexion, causing different magnitudes of joint torque to be released with varying angular speed. Recently, distinct patterns of antagonist muscle activation and joint stiffness were barely observed during collaborations (Choi et al., 2020a; Loh et al., 2020), probably because the simulation settings for power assistance were mainly based on static force control in isometric contraction.

We used a specially devised isokinetic exercise and dynamometer system to simulate the collaboration with actual power assistance on elbow joint movement. Isokinetic exercise has been used in the fields of rehabilitation and clinical assessments (Almutairi et al., 2023; Amiridis, 1996; Marchant et al., 2009; Sin et al., 2014). We postulated that this could facilitate power assistance simulation in terms of controlled reproduction of exercise conditions by altering joint angles and rotation speed.

This study investigated the effect of torque release, which is caused by angular movement of power assistance, on muscle activation, output of joint torque, and the perceived level of assistance. Our experimental exercise task involved serial torque transition from fully manual isometric contraction on the lever arm to isokinetic torque release by power assistance. We examined not only transient muscle activations of the biceps brachii (BB) and brachioradialis (BR) muscles as agonists of isokinetic elbow flexion and the triceps brachii (TB) muscle as an antagonist, but also the co-contraction index (CCI) of these muscle activations. For joint torque, time-dependent change and variability were analyzed. The variables of isometric and isokinetic exercises were set as the baseline torque and angular speed, respectively. We hypothesized that muscular and torque responses during isokinetic torque release would be dependent on the exercise variables, which are related with the speed of robotic arm movement and the torque magnitude required to initiate power assistance. Specifically, we also hypothesized that the patterns of antagonist muscle activation would vary during torque release.

## Methods

### Participants

**Figure 1:**
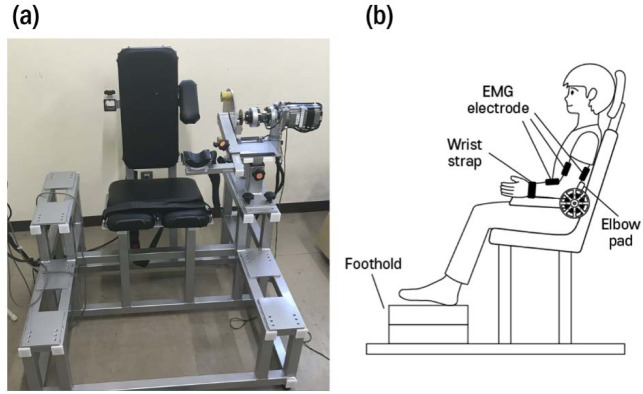
(a) An isokinetic dynamometer system and (b) a schematic illustration of the experimental setup from the sagittal plane of view.

Ten male participants volunteered for this study (age: 23.8 ± 2.7 years, body height: 176.5 ± 4.4 cm, body mass: 67.4 ± 9.8 kg). Only participants taller than 170 cm were recruited because of the seat dimension limitation of the isokinetic dynamometer system (S-17025; Takei Scientific Instruments Co., Ltd.) (Figure 1a) used in this study. No participant had a current or a previous history of upper limb functional impairment. All participants were right-handed, as ascertained using the Edinburgh Handedness Inventory (Oldfield, 1971). This studycomplied with the tenets of the Declaration of Helsinki and was approved by the Research Ethics Committee of the Faculty of Design of the Kyushu University (approval number: 428; approval date: 29 July 2021).

For sample size calculation, a priori power analysis was conducted using G*Power (v 3.1.9.7, University of Dusseldorf, Dusseldorf, Germany) based on recently published data from a previous study (Choi et al., 2020b) involving a similar population (i.e., male undergraduate and graduate students [n = 11]) and similar tasks of muscle force release. To satisfy the usual recommendation of 80% power (1–β error probability) at the 5% level of α error needed to conclude that a result is significant for the major dependent variable of torque variability, a minimum sample size of n = 10 was required.

### Experimental Setup

The experimental setup is illustrated in Figure 1b. Participants were seated upright in the isokinetic dynamometer system chair with their feet firmly placed on a foothold, the height of which could be adjusted according to their leg length. Their non-dominant forearm was positioned parallel to the lever arm of the system in neutral rotation. Their wrist and elbow were firmly placed on the wrist pad of the lever arm and the elbow pad of the chair, respectively. The length of the lever arm was adjusted according to their forearm length. The upper arm and forearm were then positioned at an elbow angle of 90°. A wrist strap was attached at the level of the styloid process to firmly connect the lever arm and the wrist. Participants were able to generate elbow joint torque on the lever arm when they steadily pulled the lever arm upwards.

### Power Assistance Task

Prior to the power assistance task, the maximum voluntary torque (MVT) value generated during isometric elbow flexion at a right angle was measured for each participant, using the dynamometer system. It was calculated from the maximum value of three 5-s trials, with at least 60 s of rest provided in between. It was used to calculate submaximal target torque ranges for each participant.

**Figure 2:**
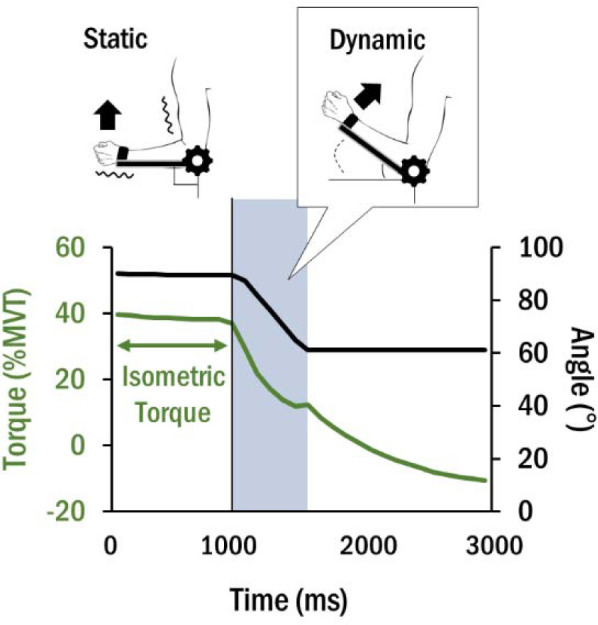
A representative configuration of the power assistance task for the exercise condition of GIT/60°•s^−1^. The black line represents the change of the lever arm angle caused by power assistance. The green line represents the change of manual torque applied on the lever arm. Before the operation of the lever arm, isometric joint torque is applied at 90° on the elbow joint. It is released along with the rotation of the lever arm to 60° of the elbow joint.

The main task for simulating power assistance involved two submaximal torque control phases: 10–30 %MVT as lower isometric torque (LIT) and 30–50 %MVT as greater isometric torque (GIT). During the first baseline phase, the participant was instructed to exert and maintain upward isometric torque of either LIT or GIT at 90° of elbow flexion. Once the torque was successfully maintained in this range for 3 s, the lever arm was rotated to an elbow angle of 60° (i.e., 30° displacement) with a constant speed of either 60°•s^−1^ or 90°•s^−1^. This resulted in torque release and mechanically assisted isokinetic elbow flexion (i.e., power assistance). For example, once 100 ms had passed after power assistance, the elbow angle was rotated to 84° under the speed condition of 60°•s^−1^ and to 81° under the condition of 90°•s^−1^. Figure 2 illustrates the configuration of the power assistance task and the angle change of the elbow joint. Although the joint torque was released, participants were asked to maintain the torque set for the first phase for as long as possible until the lever arm was fully rotated. Each trial was repeated five times.

No sensory feedback from the dynamometer was provided to participants. They were only asked to focus on a red circle (diameter: 10 cm), marked 2 m away from them at the eye level, during the experimental session to minimize possible distractions. A familiarization session was conducted before the main experimental session in order to train participants to maintain the baseline torque without feedback. Participants had to repeat ten trials of the power assisted task.

### Measurements

The torque output applied to the lever arm was gravity-corrected and measured by a torque sensor mounted between the lever arm and a servo motor of the dynamometer system. The EMG amplitude of the BB, BR, and TB muscles was obtained using surface bipolar active EMG electrodes (BA-U410m; Nihon Santaku, Osaka, Japan). After site preparation, the electrodes were placed in line with the direction of the muscle fiber, following the SENIAM recommendations (Hermens et al., 2000). A ground electrode was placed on the left acromion. EMG signals were 1,000 times amplified using a bio-instrumentation amplifier (BA1104m; Nihon Santeku), as well as band-pass filtered (10–500 Hz) (Ali et al., 2015), and full-wave rectified. The data from the EMG and the dynamometer were A/D converted by PowerLab 16/30 (ADInstruments; Dunedin, New Zealand) at 2 KHz, and stored in a computer.

The values of maximal voluntary contractions (MVCs) were measured for each muscle, to normalize EMG amplitudes between participants (%MVC). The MVCs of the elbow flexor muscles (BB and BR) were measured simultaneously with the MVT trials, while the MVC of the TB muscle was obtained in separate trials. During the trial, strong verbal encouragement was provided to obtain maximal contractions. Each MVC trial lasted 5 s with 60 s of rest in between. The mean rectified amplitude value over the middle 3 s of MVC trials was obtained for EMG normalization.

To examine the perceived level of power assistance by lever arm rotation, a 10-point scale was created, with a rating of 1 denoting very light assistance and 10 denoting maximal assistance. It was rated immediately after each trial by participants responding to the question “How much assistance did you feel?”.

### Procedures

Four sequential sessions were completed in this study: (1) physical measurement and electrode placement, (2) MVT and MVC measurements, (3) familiarization trials, and (4) the main experiment of power assistance simulation. Once the MVT and MVC values were obtained, participants were trained to maintain both torque ranges without any external feedback. During the main experiment, participants performed four exercise conditions with different torque baselines and rotation speeds. Their order was completely randomized to minimize the carry-over effect. Five minutes of rest were given when all the trials of each condition were finished. The sessions lasted 40 min.

### Data Analysis

Three time epochs of 100 ms were evaluated based on the output of the power assistance task: *E*_0_ representing pre-release (200–100 ms to the moment of lever arm rotation), *E*_1_ representing the torque release for 0–100 ms, 0 representing the moment of lever arm rotation and *E*_2_ for 100–200 ms. For these three epochs, the normalized muscle activation was obtained and the standard deviation (SD) of the torque output was calculated as the torque variability. We also estimated simultaneous contraction of multiple muscles by calculating the CCI based on the equation (1) (Chalard et al., 2020), where the EMG extensor is the normalized EMG amplitude of the TB, and the EMG flexors are the average of normalized EMG amplitude of the BB and BR.


$$\text{CCI(\%)= 2x }\frac{EMG_{Extensor}}{EMG_{Extensor}+EMG_{Flexors}}\text{x100 (1)}$$

The data were processed using LabChart 8 (ADInstruments) and Excel (Microsoft, Redmond, WA, USA).

### Statistical Analysis

A linear mixed model was used to examine the effects of three factors: time epoch for the pre-release and release phases, baseline torque in the pre-release phase (LIT and GIT), and the rotation speed of the lever arm (60°•s^−1^ and 90°•s^−1^) on objective measurements. This model was chosen because the relationship between the time epoch and baseline torque could violate the independence assumption of repeated measures analysis of variance (ANOVA). The three factors were used as fixed effects in the model, while the participant identification number was used as the random effect. Because subjective ratings were obtained after the completion of one exercise condition, a two-way repeated measures ANOVA was applied for the perceived assistance level. The Shapiro-Wilk test confirmed the normality of the collected data. When a statistically significant effect was observed, Bonferroni-corrected pairwise comparison was conducted as a post-hoc test. SPSS Statistics 27.0 (IBM, Research Triangle Park, NC, USA) and modules in R (v4.3.0; R Foundation for Statistical Computing, Vienna, Austria) were used for statistical analyses. Statistical significance was set as α = 0.05. Partial eta squared ($\eta_{p}^{2}$) was reported as a measure of the effect size using the ‘effectsize’ package in R. All values are presented as mean ± SE.

## Results

The average MVT measured during isometric elbow flexion was 37.90 ± 2.92 N•m.Figure 2 demonstrates averaged time series output of torque and muscle activation in the three muscles for 3,000 ms, with 1,000 ms as the moment of operating power assistance. The isometric torque was maintained and released when the lever arm rotated.

### Torque Output and Variability

A significant main effect of the time epoch was observed in torque output (F(2,99) = 144.27, *p*< 0.01, $\eta_{p}^{2}$ = 0.74), which gradually decreased over time (E0: 30.05 ± 1.46 %MVT; E1: 20.38 ± 1.35 %MVT; E2: 14.24 ± 1.29 %MVT). Significant main effects of baseline torque and rotation speed were also identified (F(1,99) = 319.79, *p*< 0.01, $\eta_{p}^{2}$ = 0.76; F(1,99) = 14.26, *p*< 0.01, $\eta_{p}^{2}$ = 0.13), with GIT and slower speed demonstrating greater torque output. Significant interactions between (1) time epoch and baseline torque and (2) time epoch and rotation speed were observed (F(2,99) = 5.02, *p*< 0.01, $\eta_{p}^{2}$ = 0.09; F(2,99) = 3.36, *p*< 0.05, $\eta_{p}^{2}$ = 0.06). The corresponding post-hoc tests revealed that baseline torque and rotation speed did not affect torque output in E0. The interaction between baseline torque and rotation speed as well as a three-way interaction were not significant.

For the variability in torque output, we observed significant main effects of the time epoch (F(2,99) = 307.95, *p*< 0.01, $\eta_{p}^{2}$ = 0.86), baseline torque (F(1,99) = 61.76, *p*< 0.01, $\eta_{p}^{2}$ = 0.38), and rotation speed (F(2,99) = 30.58, *p*< 0.01, $\eta_{p}^{2}$ = 0.38). The corresponding post-hoc tests demonstrated that there was greater variability in E1 (2.70 ± 0.12) and E2 (2.08 ± 0.17), compared to E0 (0.99 ± 0.03). Greater variability was also observed for GIT (2.01 ± 0.09) and faster rotation speed (1.92 ± 0.08) compared with their counterparts (LIT: 1.37 ± 0.08; slower: 1.48 ± 0.06). A two-way interaction between the time epoch and baseline torque was observed to be significant (F (2,99) = 8.66, *p*< 0.01, $\eta_{p}^{2}$ = 0.15), and the interaction between the time epoch and rotation speed was also significant (F (2,99) = 7.03, *p*< 0.01, $\eta_{p}^{2}$ = 0.12). Post-hoc tests for these interactions demonstrated that GIT and faster rotation speed had greater variability in E1 and E2, while E0 remained non-significant. The effects of the other terms were not significant.

### EMG Amplitude

Figure 4 illustrates changes in rectified EMG for the BB, BR, and TB muscles over time. Similar to torque output, muscle activation decreased after power assistance. The averaged values in each time epoch were statistically evaluated.

**Figure 3:**
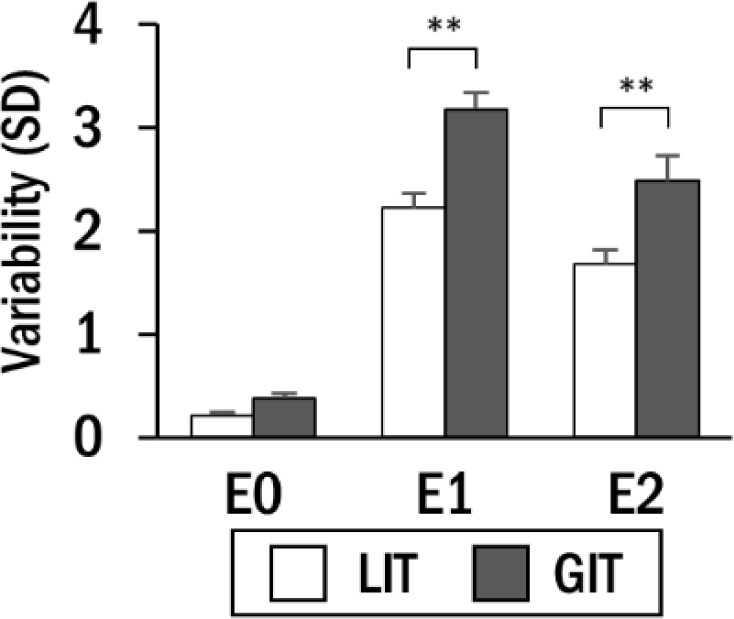
Average torque variability by time epoch for each baseline torque. Asterisks indicate significant difference between baseline torque (** *p*< 0.01).

**Figure 4:**
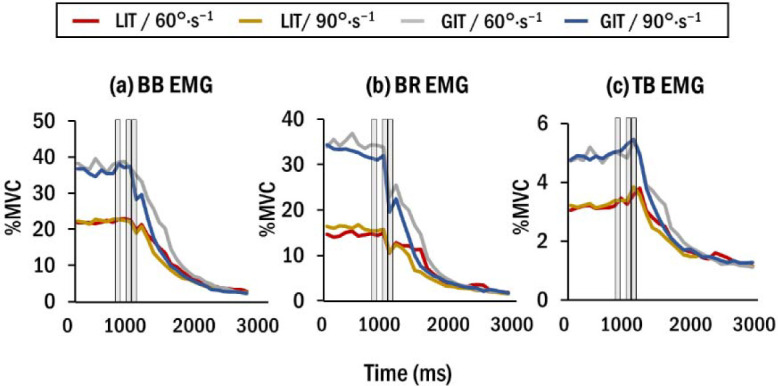
Time-series graphs of rectified EMG amplitudes of (a) BB, (b) BR, and (c) TB muscles. Shaded areas represent the three observed time epochs of E0, E1, and E2, respectively. Data are averaged across all participants (n = 10). For convenient visualization, the sampling frequency is set to 10 Hz.

**Figure 5:**
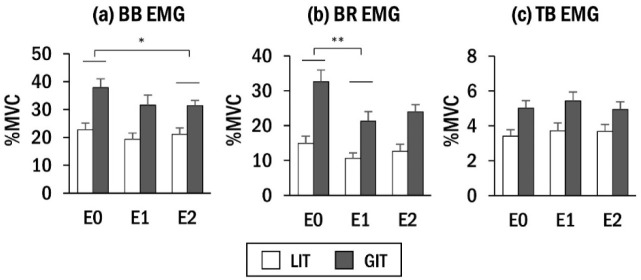
Average EMG amplitude in (a) BB (b) BR, and (c) TB muscles by time epoch for each baseline torque. Asterisks indicate significant difference between time epochs (* *p*< 0.05; ** *p*< 0.01).

The results of BB muscle activation (Figure 5a) demonstrated significant main effects of the time epoch (F(2,99) = 5.60, *p*< 0.01,$\eta_{p}^{2}$ = 0.10), with E2 (26.24 ± 1.86 %MVC) exhibiting a lower EMG amplitude compared with E0 (30.30 ± 2.25 %MVC). Baseline torque had a significant effect (F(1,99) = 98.21, *p*< 0.01, $\eta_{p}^{2}$ = 0.50), with GIT (33.62 ± 2.46 %MVC) demonstrating greater EMG amplitude compared with LIT (21.05 ± 2.13 %MVC). The other terms including rotation speed did not demonstrate significant effects.

A significant main effect of the time epoch on BR muscle activation (Figure 5b) was observed (F(2,99) = 13.49, *p*< 0.01, $\eta_{p}^{2}$ = 0.21), with E1 (15.92±1.73 %MVC) demonstrating a lower EMG amplitude than E0 (23.77 ± 2.23 %MVC). A significant main effect was also observed for baseline torque (F(1,99) = 109.66, *p*< 0.01, $\eta_{p}^{2}$ = 0.53), with GIT (25.96 ± 2.41 %MVC) indicating greater EMG amplitude than LIT (12.70 ± 1.64 %MVC). The interaction between the time epoch and baseline torque was significant (F(2,99) = 3.13, *p*< 0.05, $\eta_{p}^{2}$ = 0.06). Post-hoc tests revealed a significant difference in the EMG amplitude between E0 (32.63 ± 3.34 %MVC) and E1 (21.25 ± 2.76 %MVC) for GIT, while no significant difference was shown between E0 (14.92 ± 2.04 %MVC) and E1 (10.59 ± 1.59 %MVC) for the LIT (*p* = 0.15). The other terms were not significant.

For TB muscle activation, the main effect of the time epoch was not significant (*p* = 0.12) (Figure 5c). Baseline torque had a significant effect (F(1,99) = 107.28, *p*< 0.01, $\eta_{p}^{2}$ = 0.52), with GIT (5.13 ± 0.43 %MVC) indicating greater EMG amplitude than LIT (3.60 ± 0.37 %MVC). No other significant effects were observed.

### CCI

**Figure 6:**
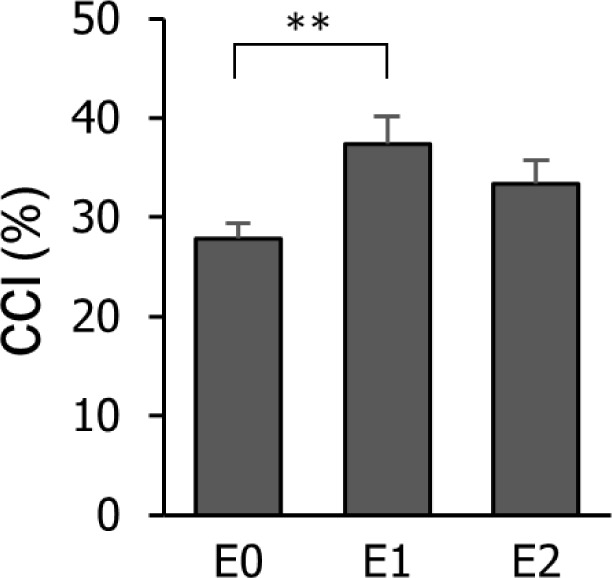
Average CCI by time epoch. Asterisks indicate significant difference (** *p*< 0.01).

The main effect of the time epoch was significant (F(2,99) = 31.54, *p*< 0.01, $\eta_{p}^{2}$ = 0.39), with E1 (37.43 ± 2.70 %) showing greater CCI compared with E0 (27.90 ± 1.50 %) (Figure 6).Baseline torque had a significant effect (F(1,99) = 30.16, *p*< 0.01, $\eta_{p}^{2}$ = 0.23), with GIT (35.62 ± 2.04 %) demonstrating greater CCI compared to LIT (30.21 ± 2.05%). The other terms were not significant.

### Perceived Assistance Level

The main effect of baseline torque was significant (F(1,9) = 12.85, *p*< 0.01, $\eta_{p}^{2}$ = 0.59). The post-hoc test indicated a higher perceived assistance level in LIT (7.65 ± 0.30) than in GIT (6.20 ± 0.42). No other significant differences were found.

## Discussion

This study examined how muscle activation and torque output altered during elbow flexion when the baseline torque of greater and lower isometric contraction was released by power assistance with faster and slower rotation speeds. The responses in the pre-release phase (E0) were not affected by exercise conditions. However, different activation patterns of the agonist and antagonist muscles were observed in the release phase. While greater variability and a lower level of perceived assistance were observed with the release of greater baseline torque, rotation speed did not affect muscle activation of the related muscles.

### Changes in the Pre-Release Phase of Torque

Participants were trained to operate the lever arm when the torque output was maintained in the LIT or GIT range for 3 s during the familiarization session. Hence, we noted the possibility of anticipatory postural adjustment due to the increased activation of the agonist or antagonist muscles before torque release (Holmes and Keir, 2012; Koike and Yamada, 2007). There is evidence that the central nervous system increases joint stiffness prior to and after perturbation if its timing can be anticipated (Dupeyron et al., 2010). A faster rotation speed of the lever arm was expected to influence the activation patterns of the TB muscle because such a predictive response of the antagonist would take place according to the magnitude of perturbation (Franklin et al., 2003). However, there was no difference in muscle activation in the BB, BR, and TB muscles across exercise conditions in the pre-release time epoch. Although the requirements to operate power assistance were learned, participants could have trouble anticipating the exact timing of the lever arm operation in a timely manner without any sensory feedback, which had been used in previous studies (Koike and Yamada, 2007; Petersen et al., 2009). This is also in line with previous findings that demonstrated no muscle activation changes prior to unexpected perturbation (Holmes and Keir, 2012).

### Changes in the Release Phase of Torque

Similar to previous studies on isometric force control, greater variability was observed when torque was released from a higher baseline (GIT) (Choi et al., 2020a, 2019). Hence, if the magnitude of the initial manual control is greater, the torque mismatch between the joint inertia and power assistance could prevent the smooth transition from manual control to collaboration. The variability was further increased when power assistance was provided at a higher speed, which demonstrates that the speed-accuracy trade-off in target-directed movements (Elliott et al., 2004; Plamondon and Alimi, 1997) can also be applied during the collaboration. On the other hand, the increased torque variability in E1 decreased in E2 in the period after 100 ms regardless of rotation speed and baseline torque, demonstrating that the upper-limb musculature is capable of damping instantly increased torque variability caused by power assistance.

BB and BR muscle activation reduced immediately with the initiation of power assistance. This indicates that these muscles play a leading role in releasing joint torque for collaboration as agonist muscles in elbow flexion. Several studies have also demonstrated that power assistance is beneficial in reducing activation in the agonist muscle of the related joint(Huysamen et al., 2018; Hwang et al., 2021). In particular, the response of the BR muscle is more sensitive to power assistance as the onset of its EMG amplitude reduction was faster than that of the BB EMG amplitude. This could be because the wrist position was maintained as neutral throughout the trial, where the BR muscle is known to have greater activation during elbow flexion with the wrist in the neutral position (Bressel et al., 2001).A neutral grip is suggested for lifting tasks in industrial settings in order to optimize elbow flexion torque and steadiness (O’Connell et al., 2021; Zehr et al., 2020). Future studies for power assistance on elbow flexion may highlight BR activation in addition to the BB.

In contrast, our results of TB muscle activation and CCI support the hypothesis that the activation patterns of the TB muscle are distinct from those of agonist muscles as they did not decrease from pre-release to 200 ms after release. Such patterns could reflect that the joint stiffness strategy to maintain postural stability can be applied for sudden joint torque release by power assistance (Hasan, 2005; Latash, 1992) as well as for sudden joint loading (Holmes and Keir, 2012).This is interesting because our previous studies on force release by visually guided force tracking (Choi et al., 2019) and mechanical assistance (Choi et al., 2020a) during isometric elbow flexion did not find distinct patterns of muscle co-contraction although EMG amplitude of the elbow flexor was reduced. Therefore, the need for postural stabilization mechanisms by the co-contraction of the antagonist muscle could increase when transient changes in the joint angle and torque release take place due to power assistance. On the other hand, rotation speed did not significantly affect the changes of muscle activation patterns, while torque output and its variability were affected by both rotation speed and baseline torque over time. This suggests that the baseline torque required for initiating the operation of power assistance should be given priority to the rotation speed of the robotic arm when it comes to the design of power assistance considering human muscular characteristics.

### Perceived Assistance

Previous studies have demonstrated that perceived effort was reduced while using power assistance (Huysamen et al., 2018; Rashedi et al., 2014) or after isometric force release (Choi et al., 2019). However, how humans perceive the level of assistance could also be important for successful collaboration. Our results demonstrate that the perceived assistance level was relatively high in LIT and with a faster rotation speed. This is contradictory to our expectation because when a greater amount of torque is released by power assistance, a more energetic benefit should take place. One possibility is that when the baseline torque is low, the proportion of manual control in the collaboration is reduced. Specifically, a faster rotation speed of the lever arm at LIT could allow participants to lose control of the collaboration to a level close to a complete delegation. Thus, participants could have perceived more assistance as a result of reduced operability. Conversely, when the baseline torque is greater, a relatively high level of muscle activation is continued while maintaining higher operability during torque release. These results imply major conflicting perspectives derived from human and power assistance collaboration studies. The basic utility of power assistance is to reduce joint torque and muscle activation, while humans should remain as an operator of collaboration (Choi et al., 2020a; Novakovic and Sanguineti, 2011). Although the current study used general inquiry to determine the level of perceived assistance, future studies should focus on an in-depth relationship of these two independent dimensions of perceived benefit and operability.

## Practical Implications

In order to enhance collaboration with power assistance, the exercise variables such as the speed of the lever arm (i.e., robotic arm) and the joint torque required for operating power assistance should be carefully determined at the design stage. The current results showed that muscle co-contraction increased instantly after the torque release, regardless of the rotation speed (60–90°•s^−1^). Hence, the potential benefit of slowing the rotation speed of the lever arm in lowering the muscle co-contraction ratio has not been observed in this study.

## Conclusions

This study analyzed transient muscle activation patterns in isokinetic elbow flexion when isometric torque was released by power assistance. Our findings demonstrated distinct patterns of EMG amplitude by agonist and antagonist muscles. TB EMG amplitude remained relatively consistent although BB and BR EMG amplitude was reduced immediately after power assistance. The perceived assistance level was low when a greater level of baseline torque was released. Further investigation is needed to determine whether such patterns alter with collaboration training of a relatively long period and with sensory feedback provided prior to power assistance, so that the exact perturbation timing can be accurately anticipated.

## References

[ref1] Ali, M. A., Sundaraj, K., Ahmad, R. B., Ahamed, N. U., Islam, M. A., & Sundaraj, S. (2015). Muscle Fatigue in the Three Heads of the Triceps Brachii during a Controlled Forceful Hand Grip Task with Full Elbow Extension Using Surface Electromyography. Journal of Human Kinetics, 46(1), 69–76. 10.1515/hukin-2015-0035 26240650 PMC4519223

[ref2] Almutairi, M. K., Hunter, G. R., Lein, D. H., Kim, S., Bryan, D. R., Inacio, M., Hurt, C. P., Reed, W., & Singh, H. (2023). Enhancement of Muscle Shortening Torque Preloaded with Muscle Lengthening is Joint-Specific. Journal of Human Kinetics, 87, 11–21. 10.5114/jhk/161729 37229413 PMC10203843

[ref3] Amiridis, I. G. (1996). Co-activation and tension-regulating phenomena during isokinetic knee extension in sedentary and highly skilled humans. European Journal of Applied Physiology and Occupational Physiology, 73(1–2), 149–156. 10.1007/bf00262824 8861684

[ref4] Andrzejewska, R., Jaskólski, A., Jaskólska, A., Gobbo, M., & Orizio, C. (2014). Electromyogram features during linear torque decrement and their changes with fatigue. European Journal of Applied Physiology, 114(10), 2105–2117. 10.1007/s00421-014-2928-4 24957414

[ref5] Bressel, E., Bressel, M., Marquez, M., & Heise, G. D. (2001). The effect of handgrip position on upper extremity neuromuscular responses to arm cranking exercise. In Journal of Electromyography and Kinesiology (Vol. 11). www.elsevier.com/locate/jelekin 10.1016/s1050-6411(01)00002-5 11532600

[ref6] Buchanan, T. S., Rovai, G. P., & Rymer, W. Z. (1989). Strategies for muscle activation during isometric torque generation at the human elbow. Journal of Neurophysiology, 62(6), 1201–1212. 10.1152/jn.1989.62.6.1201 2600619

[ref7] Chalard, A., Belle, M., Montané, E., Marque, P., Amarantini, D., & Gasq, D. (2020). Impact of the EMG normalization method on muscle activation and the antagonist-agonist co-contraction index during active elbow extension: Practical implications for post-stroke subjects. Journal of Electromyography and Kinesiology, 51. 10.1016/j.jelekin.2020.102403 32105912

[ref8] Choi, J., Yeoh, W. L., Loh, P. Y., & Muraki, S. (2019). Force and electromyography responses during isometric force release of different rates and step-down magnitudes. Human Movement Science, 67(April), 102516. 10.1016/j.humov.2019.102516 31539754

[ref9] Choi, J., Yeoh, W. L., Loh, P. Y., & Muraki, S. (2020). Motor performance patterns between unilateral mechanical assistance and bilateral muscle contraction. International Journal of Industrial Ergonomics, 80(November), 103056. 10.1016/j.ergon.2020.103056

[ref10] Choi, J., Yeoh, W. L., Matsuura, S., Loh, P. Y., & Muraki, S. (2020). Effects of mechanical assistance on muscle activity and motor performance during isometric elbow flexion. Journal of Electromyography and Kinesiology, 50(October 2019), 102380. 10.1016/j.jelekin.2019.102380 31841884

[ref11] Dupeyron, A., Perrey, S., Micallef, J. P., & Pélissier, J. (2010). Influence of back muscle fatigue on lumbar reflex adaptation during sudden external force perturbations. Journal of Electromyography and Kinesiology, 20(3), 426–432. 10.1016/j.jelekin.2009.05.004 19595613

[ref12] Elliott, D., Hansen, S., Mendoza, J., & Tremblay, L. (2004). Learning to optimize speed, accuracy, and energy expenditure: A framework for understanding speed-accuracy relations in goal-directed aiming. Journal of Motor Behavior, 36(3), 339–351. 10.3200/JMBR.36.3.339-351 15262629

[ref13] Franklin, D. W., Osu, R., Burdet, E., Kawato, M., & Milner, T. E. (2003). Adaptation to Stable and Unstable Dynamics Achieved by Combined Impedance Control and Inverse Dynamics Model. Journal of Neurophysiology, 90(5), 3270–3282. 10.1152/jn.01112.2002 14615432

[ref14] Galle, S., Derave, W., Bossuyt, F., Calders, P., Malcolm, P., & De Clercq, D. (2017). Exoskeleton plantarflexion assistance for elderly. Gait and Posture, 52, 183–188. 10.1016/j.gaitpost.2016.11.040 27915222

[ref15] Hasan, Z. (2005). The human motor control system’s response to mechanical perturbation: Should it, can it, and does it ensure stability? Journal of Motor Behavior, 37(6), 484–493. 10.3200/JMBR.37.6.484-493 16280319

[ref16] Hermens, H. J., Freriks, B., Disselhorst-Klug, C., & Rau, G. (2000). Development of recommendations for SEMG sensors and sensor placement procedures. Journal of Electromyography and Kinesiology, 10(5), 361–374. 10.1016/S1050-6411(00)00027-4 11018445

[ref17] Holmes, M. W. R., & Keir, P. J. (2012). Posture and hand load alter muscular response to sudden elbow perturbations. Journal of Electromyography and Kinesiology, 22(2), 191–198. 10.1016/j.jelekin.2011.11.006 22137184

[ref18] Huysamen, K., Bosch, T., de Looze, M., Stadler, K. S., Graf, E., & O’Sullivan, L. W. (2018). Evaluation of a passive exoskeleton for static upper limb activities. Applied Ergonomics, 70(July), 148–155. 10.1016/j.apergo.2018.02.009 29866305

[ref19] Hwang, J., Kumar Yerriboina, V. N., Ari, H., & Kim, J. H. (2021). Effects of passive back-support exoskeletons on physical demands and usability during patient transfer tasks. Applied Ergonomics, 93(August 2020), 103373. 10.1016/j.apergo.2021.103373 33516046

[ref20] Iranzo, S., Piedrabuena, A., Iordanov, D., Martinez-Iranzo, U., & Belda-Lois, J. M. (2020). Ergonomics assessment of passive upper-limb exoskeletons in an automotive assembly plant. Applied Ergonomics, 87. 10.1016/j.apergo.2020.103120 32310110

[ref21] Kiguchi, K., Tanaka, T., & Fukuda, T. (2004). Neuro-fuzzy control of a robotic exoskeleton with EMG signals. IEEE Transactions on Fuzzy Systems, 12(4), 481–490. 10.1109/TFUZZ.2004.832525

[ref22] Koike, T., & Yamada, N. (2007). Anticipation of elbow joint perturbation shortens the onset time of the reflex EMG response in biceps brachii and triceps brachii. Neuroscience Letters, 412(1), 56–61. 10.1016/j.neulet.2006.10.058 17194539

[ref23] Latash, M. L. (1992). Virtual trajectories, joint stiffness, and changes in the limb natural frequency during single-joint oscillatory movements. Neuroscience, 49(1), 209–220. 10.1016/0306-4522(92)90089-K 1407547

[ref24] Lee, H., Lee, B., Kim, W., Gil, M., Han, J., & Han, C. (2012). Human-robot cooperative control based on pHRI (Physical human-robot interaction) of exoskeleton robot for a human upper extremity. International Journal of Precision Engineering and Manufacturing, 13(6), 985–992. 10.1007/s12541-012-0128-x

[ref25] Lewis, G. N., MacKinnon, C. D., Trumbower, R., & Perreault, E. J. (2010). Co-contraction modifies the stretch reflex elicited in muscles shortened by a joint perturbation. Experimental Brain Research, 207(1–2), 39–48. 10.1007/s00221-010-2426-9 20878148 PMC3045052

[ref26] Loh, P. Y., Hayashi, K., Nasir, N., & Muraki, S. (2020). Changes in Muscle Activity in Response to Assistive Force during Isometric Elbow Flexion. Journal of Motor Behavior, 52(5), 634–642. 10.1080/00222895.2019.1670128 31571525

[ref27] Marchant, D. C., Greig, M., & Scott, C. (2009). Attentional Focusing Instructions Influence Force Production and Muscular Activity During Isokinetic Elbow Flexions. Journal of Strength and Conditioning Research, 23(8), 2358–2366. 10.1519/JSC.0b013e3181b8d1e5 19826287

[ref28] Novakovic, V., & Sanguineti, V. (2011). Adaptation to constant-magnitude assistive forces: Kinematic and neural correlates. Experimental Brain Research, 209(3), 425–436. 10.1007/s00221-011-2573-7 21305377

[ref29] O’Connell, D. G., Cooper, K. A., Richeson, S. M., Moeller, M. B., Stephens, J. W., & Key, D. A. J. (2021). The effect of forearm position on elbow flexion strength in nursing, occupational, and physical therapy students. Work, 69(2), 403–409. 10.3233/WOR-213486 34092689

[ref30] Orizio, C., Baruzzi, E., Gaffurini, P., Diemont, B., & Gobbo, M. (2010). Electromyogram and force fluctuation during different linearly varying isometric motor tasks. Journal of Electromyography and Kinesiology, 20(4), 732–741. 10.1016/j.jelekin.2010.03 20395156

[ref31] Papla M., Latocha A., Grzyb W., Golas A. Relationship between lower limb power output, sprint and change of direction performance in soccer players. Balt J Health Phys Act. 2022;14(3):Article3. 10.29359/BJHPA.14.3.03.005

[ref32] Peternel, L., Noda, T., Petrič, T., Ude, A., Morimoto, J., & Babič, J. (2016). Adaptive control of exoskeleton robots for periodic assistive behaviours based on EMG feedback minimisation. PLoS ONE, 11(2), 1–26. 10.1371/journal.pone.0148942 PMC4755662 26881743

[ref33] Petersen, T. H., Rosenberg, K., Petersen, N. C., & Nielsen, J. B. (2009). Cortical involvement in anticipatory postural reactions in man. Experimental Brain Research, 193(2), 161–171. 10.1007/s00221-008-1603-6 18956177

[ref34] Plamondon, R., & Alimi, A. M. (1997). Speed/accuracy trade-offs in target-directed movements. Behavioral and Brain Sciences, 20(2), 279–303. 10.1017/S0140525X97001441 10096999

[ref35] Rashedi, E., Kim, S., Nussbaum, M. A., & Agnew, M. J. (2014). Ergonomic evaluation of a wearable assistive device for overhead work. Ergonomics, 57(12), 1864–1874. 10.1080/00140139.2014.952682 25183258

[ref36] Sin, M., Kim, W. S., Park, D., Min, Y. S., Kim, W. J., Cho, K., & Paik, N. J. (2014). Electromyographic analysis of upper limb muscles during standardized isotonic and isokinetic robotic exercise of spastic elbow in patients with stroke. Journal of Electromyography and Kinesiology, 24(1), 11–17. 10.1016/j.jelekin.2013.10.002 24290983

[ref37] Sylla, N., Bonnet, V., Colledani, F., & Fraisse, P. (2014). Ergonomic contribution of ABLE exoskeleton in automotive industry. International Journal of Industrial Ergonomics, 44(4), 475–481. 10.1016/j.ergon.2014.03.008

[ref38] Zehr, J. D., Carnegie, D. R., Welsh, T. N., & Beach, T. A. C. (2020). A comparative analysis of lumbar spine mechanics during barbell-and crate-lifting: implications for occupational lifting task assessments. International Journal of Occupational Safety and Ergonomics, 26(1), 1–8. 10.1080/10803548.2018.1439872 29436289

